# Effects of Annealing Temperature on Optical Band Gap of Sol-gel Tungsten Trioxide Films

**DOI:** 10.3390/mi9080377

**Published:** 2018-07-30

**Authors:** Guanguang Zhang, Kuankuan Lu, Xiaochen Zhang, Weijian Yuan, Muyang Shi, Honglong Ning, Ruiqiang Tao, Xianzhe Liu, Rihui Yao, Junbiao Peng

**Affiliations:** Institute of Polymer Optoelectronic Materials and Devices, State Key Laboratory of Luminescent Materials and Devices, South China University of Technology, Guangzhou 510640, China; msgg-zhang@mail.scut.edu.cn (G.Z.); mskk-lu@mail.scut.edu.cn (K.L.); mszhang_xc@mail.scut.edu.cn (X.Z.); 201430320366@mail.scut.edu.cn (W.Y.); 201430320229@mail.scut.edu.cn (M.S.); 201510102158@mail.scut.edu.cn (R.T.); msliuxianzhe@mail.scut.edu.cn (X.L.); psjbpeng@scut.edu.cn (J.P.)

**Keywords:** optical band gap, tungsten trioxide film, annealing temperature, electrochromism

## Abstract

Tungsten trioxide (WO_3_) is a wide band gap semiconductor material that is used as an important electrochromic layer in electrochromic devices. In this work, the effects of the annealing temperature on the optical band gap of sol-gel WO_3_ films were investigated. X-ray Diffraction (XRD) showed that WO_3_ films were amorphous after being annealed at 100 °C, 200 °C and 300 °C, respectively, but became crystallized at 400 °C and 500 °C. An atomic force microscope (AFM) showed that the crystalline WO_3_ films were rougher than the amorphous WO_3_ films (annealed at 200 °C and 300 °C). An ultraviolet spectrophotometer showed that the optical band gap of the WO_3_ films decreased from 3.62 eV to 3.30 eV with the increase in the annealing temperature. When the Li^+^ was injected into WO_3_ film in the electrochromic reaction, the optical band gap of the WO_3_ films decreased. The correlation between the optical band gap and the electrical properties of the WO_3_ films was found in the electrochromic test by analyzing the change in the response time and the current density. The decrease in the optical band gap demonstrates that the conductivity increases with the corresponding increase in the annealing temperature.

## 1. Introduction

Tungsten trioxide (WO_3_) is an important indirect band gap semiconductor material [1]. It is used as a functional layer in the applications of gas sensors [2], photocatalysis [3], solar cells [4], water splitting [5] and electrochromism [6]. Electrochromic devices, such as smart windows [7], can meet the market demand of energy-saving devices. Since WO_3_’s electrochromic properties were found, researchers have widely studied WO_3_-based electrochromic thin films and device applications [8].

There are various choices for preparing WO_3_ films with the development of thin film technology. These include sputtering [9], chemical vapor deposition [10], vacuum evaporation [11], and sol-gel [12], among others. Currently, magnetron sputtering is a commercial technology that is used to prepare WO_3_ films due to its uniformity of film and reliability. However, the high cost issue and problems in preparing large-size devices cannot be ignored. The sol-gel method is a feasible technology for reducing the cost even, though there are still some problems at the present stage, such as film inhomogeneity and poor process repeatability, among others. With the development of new sol-gel techniques, such as inkjet printing [13], sol-gel technology is promising for commercial applications in the future.

The optical and electrical properties of WO_3_ film are related to the parameters of the sol-gel technique, such as the solvent [14], precursor [15] and annealing temperature [16], among others. In previous work, there was a significant difference in the band gap of the WO_3_ films obtained using different processes [17,18]. Therefore, it is worthwhile to launch further investigations into the relationship between band gap and the optical and electrical properties of WO_3_ films, especially in regards to electrochromic properties. In this paper, we conducted a study on the optical band gap of WO_3_ films with different annealing temperatures. The crystallinity, response time morphology and conductivity were also analyzed together. A correlation between the optical band gap and the electrical properties (conductivity) was found.

## 2. Materials and Methods

Tungsten powder (W, 99.5% metals basis, Macklin Biochemical Co. Ltd, Shanghai, China) and hydrogen peroxide (H_2_O_2_, Hydrogen peroxide 30%, Guangzhou chemical regent factory, Guangzhou, China) were mixed in a beaker with a water bath at 25 °C. After the reaction finished, an evaporative concentration treatment (at 150 °C) was conducted to remove the surplus H_2_O_2_. Finally, an appropriate anhydrous ethanol was added into the concentrated solution and the mixed solution was sealed and stirred for 3 h at 70 °C to obtain the sol-gel. A spin coating technique was used to prepare the WO_3_ films (around 80 nm) on the indium tin oxide (ITO) glass. The thickness of the WO_3_ film was optimized and controlled by the concentration of solution and spin coating parameters and it had an important influence on the electrochromic transmittance modulation ability [19]. In this work, the annealing temperature was focused on and other unrelated variables (sol concentration, spin coating parameters, substrate, electrolyte, etc.) were controlled. These as-deposited films were annealed at 100 °C, 200 °C, 300 °C, 400 °C and 500 °C for 60 min, respectively.

The crystallization of the film was analyzed by X-ray Diffraction (XRD, PANalytical Empyrean DY1577, PANalytical, Almelo, The Netherlands). The surface morphology was measured by atomic force microscopy (AFM, Being Nano-Instruments BY3000 Being Nano-Instruments, Beijing, China). The electrochromic test was performed using 0.8 mol/L of lithium perchlorate/propylene carbonate (LiClO_4_/PC) electrolyte and an electrode gap (~1 mm). The transmission spectra were measured by an Ultraviolet spectrophotometer (SHIMADZU UV2600, SHIMADZU, Tokyo, Japan), with ITO glass (Optical band gap: >4 eV) acting as a blank. The current of the electrochromic test was recorded by an electrochemical workstation (CH Instruments CHI600E, CH Instruments, Shanghai, China). The relationship between the change of transmittance and the time was measured by a micro-spectrometer (Morpho PG2000, Morpho, Shanghai, China), with ITO glass acting as a blank.

## 3. Results and Discussions

**Figure 1:**
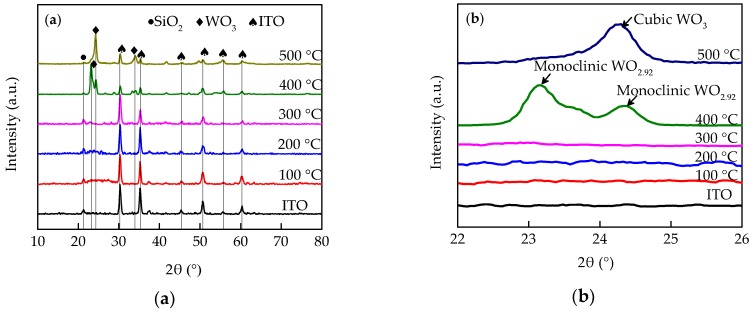
X-ray patterns of WO_3_ films annealed at different temperature. (**a**) The XRD patterns in a large range of 2θ (10° to 80°); (**b**) The XRD patterns in a small range of 2θ (22° to 26°). The amorphous WO_3_ transformed into monoclinic structure and cubic structure at 400 °C and 500 °C, respectively.

Figure 1 illustrates the X-ray patterns of the WO_3_ films that were annealed at different temperatures. The crystalline structures of these films were further analyzed using Jade 6.0 and PDF#30-1387 and PDF#41-0905. In Figure 1a, there are diffraction peaks of WO_3_ at the patterns of the WO_3_ films annealed at 400 °C and 500 °C, which demonstrate that these films transformed from amorphous to crystalline when the annealing temperature is higher than 400 °C. Furthermore, the change of crystalline structure was analyzed in Figure 1b. The characteristic diffraction peaks of WO_3_ films (400 °C) indicate that the WO_3_ films initially transform from an amorphous to a monoclinic structure. When the annealing temperature reached 500 °C, there was only one diffraction peak of the WO_3_ film in the range of 2θ (22° to 26°), which demonstrated that the monoclinic structure of the WO_3_ films turned into a cubic structure. Strictly speaking, the stoichiometric ratio of tungsten and oxygen was not fully satisfied with 1:3. Therefore, there was an oxygen vacancy which influenced the optical and electrical properties of WO_3_ films [20].

**Figure 2:**
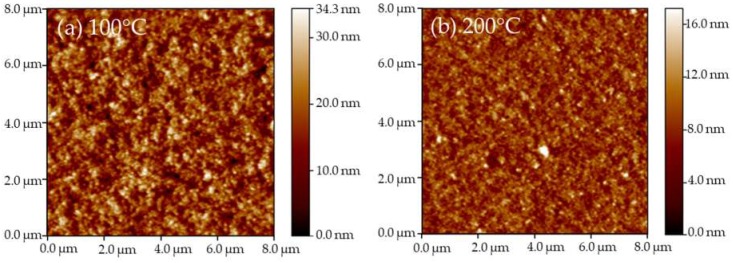

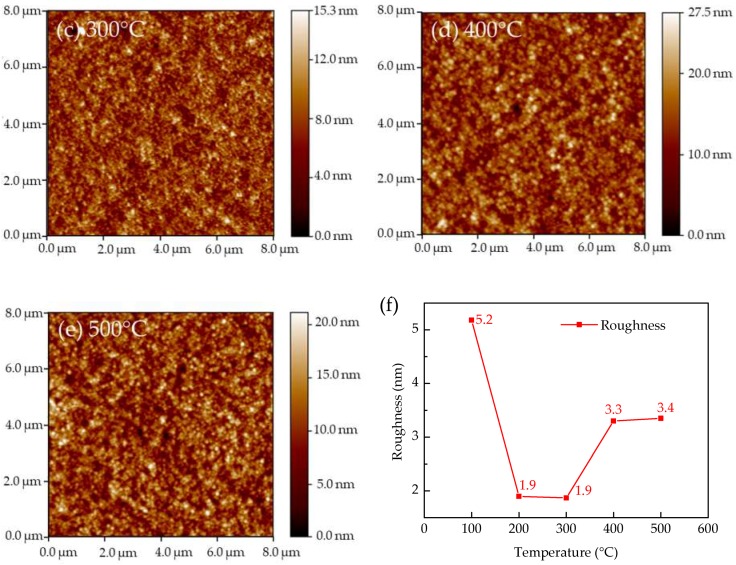
The atomic force microscope (AFM) images 8000 nm × 8000 nm) and the roughness of WO_3_ films. (**a**) 100 °C; (**b**) 200 °C; (**c**) 300 °C; (**d**) 400 °C; (**e**) 500 °C; (**f**) the roughness of WO_3_ films, which are read by the support software of AFM.

The surface morphology of these films was measured by AFM and the results are shown in Figure 2. Figure 2f shows a comparison of the roughness of these films at different annealing temperatures. The surface of the WO_3_ film that was annealed at 100 °C is rougher than other films, which is confirmed by Figure 2a and its roughness. In this work, the solvent of sol was ethanol and water, which has a boiling point of around 80 °C. The 100 °C annealing treatment can remove the solvent, but it is not enough to remove the bound water in the tungsten acid [21]. In addition, solvent evaporation can cause defects in the surface, such as pores [22], and there is not enough energy to reduce these defects during annealing treatment. Therefore, among these samples, the WO_3_ film annealed at 100 °C had the highest roughness.

The roughness of the films annealed at 200 °C and 300 °C was around 1.9 nm, which is less than that of the films (around 3.3 nm) annealed at 400 °C and 500 °C. This demonstrated that the crystalline film was rougher than the amorphous film because of its grain growth at a high temperature. The change in roughness indirectly revealed that the change in the WO_3_ film composition and crystalline structure was due to the increase in the annealing temperature, which is consistent with the results of XRD. 

The band gap of WO_3_ film can be measured and analyzed by an ultraviolet spectrophotometer. The optical band gap is distinguished from the band gap measured by other methods. According to Equation (1), the optical band gap can be calculated [23].

*αhv = A(hv − E_g_)^n^* (1)

where *α* is the absorption coefficient, which can be measured by the ultraviolet spectrophotometer; *h* is the Planck constant; *ν* is the light frequency; *A* is a proportionality constant; *E_g_* is the optical band gap; and *n* is a number which is 1/2 for the direct band gap semiconductor and 2 for the indirect band gap semiconductor. In this work, *n* is 2 because the WO_3_ was an indirect band gap semiconductor. 

To further investigate the electrochromic effects on the optical band gap of WO_3_ film, the optical band gap of WO_3_ film in a bleached state and colored state were analyzed. Electrochromism involves an electrochemical reaction, as shown in Equation (2) [24]:

(2) $${\mathrm{WO}}_{3}\left(\mathrm{colorless}\right){+\;\mathrm{xLi}}^{+}{+\;\mathrm{xe}}^{-}↔{\mathrm{Li}}_{x}{\mathrm{WO}}_{3}(\mathrm{blue})\;$$

At its bleached state, the WO_3_ film is colorless. When both Li^+^ and the electron are injected into the WO_3_ film under an applied voltage, the bleached state of WO_3_ turns into a colored state due to the generation of blue Li_x_WO_3_. 

**Figure 3:**
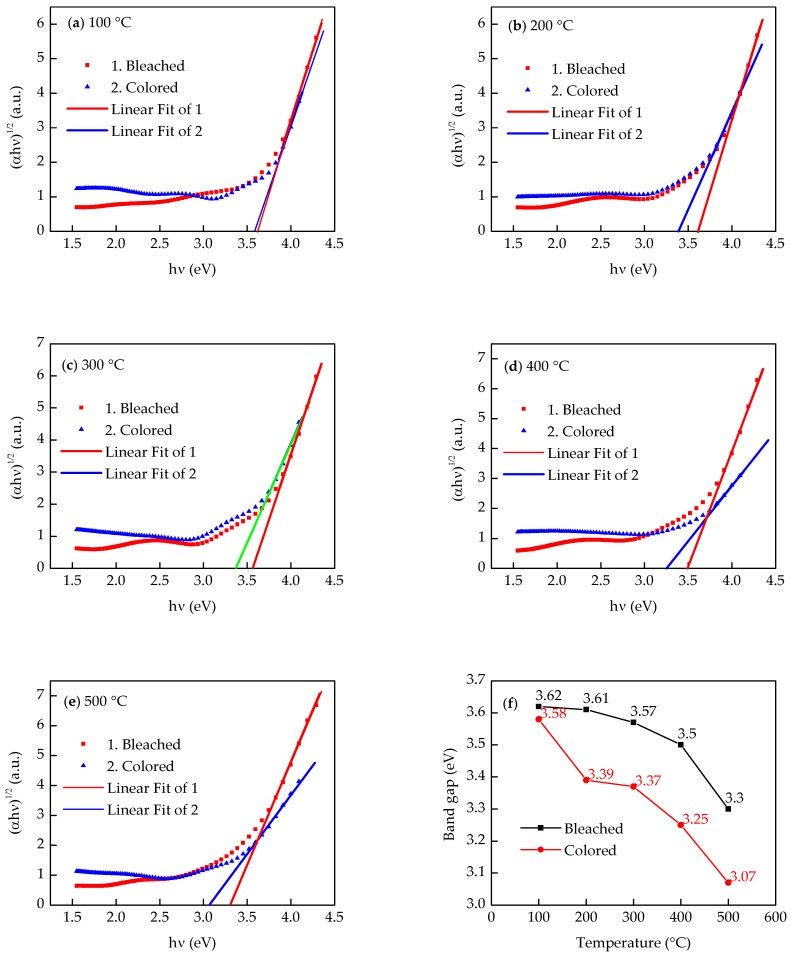
Optical band gap energy of WO_3_ films in a colored state and bleached state. (**a**) 100 °C; (**b**) 200 °C; (**c**) 300 °C; (**d**) 400 °C; (**e**) 500 °C; and (**f**) a comparison of optical band gap energy of WO_3_ films annealed at different temperature and electrochromic state (colored and bleached).

Figure 3a–e illustrates the curves of (*αhν)*^1/2^ versus the photon energy *hν*, which are calculated using the transmission spectra of the WO_3_ films in the colored state and the bleached state. *E_g_* can be extracted through the onset of the optical transitions of the WO_3_ films near the band edge, which is equal to the value of the fitting line intercepts. Figure 3f shows a comparison of the optical band gap value of the WO_3_ film that were annealed at different temperatures and electrochromic state (colored and bleached) and it indicates that the *E_g_* of bleached WO_3_ films decreases from 3.58 eV to 3.3 eV as the annealing temperature increases. Similarly, the *E_g_* of the colored WO_3_ film tends to decrease with an increased annealing temperature. In addition, the *E_g_* of all the colored WO_3_ films was less than that of their respective bleached WO_3_ films.

As for *E_g_*, which decreased when the annealing temperature increased, a reasonable explanation was that as the annealing temperature increased, the oxygen vacancies increased, which may have provided free electrons and enhanced the conductivity of the WO_3_ films [25]. 

**Figure 4:**
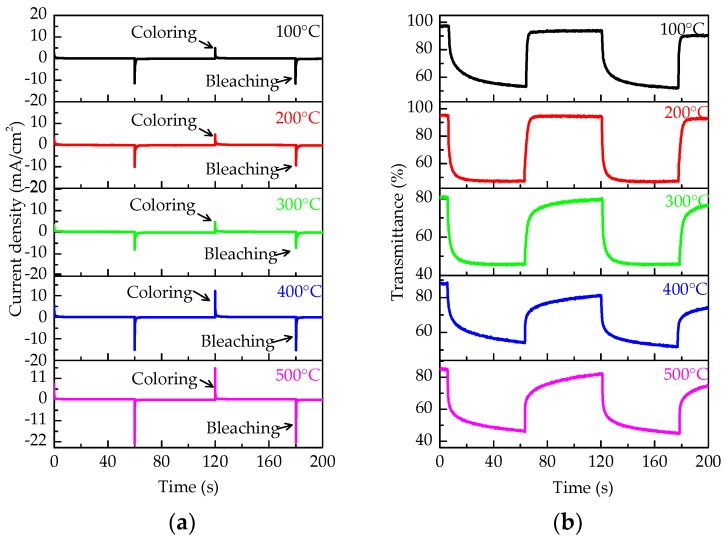
(**a**) Current change of WO_3_ films at different annealing temperature. The applied voltage was ±2.5 V and the WO_3_ films were placed in the cathode; (**b**) change of transmittance (at 600 nm) of WO_3_ films at different annealing temperature.

To further investigate the relationship between *E_g_*, conductivity, and electrochromic response time, an electrochromic test was conducted. Figure 4a,b illustrates the current density of the different WO_3_ films and the change of transmittance (at 600 nm) under ±2.5 V voltage, respectively. The peak current density of these films in the coloring process increased when the annealing temperature increased (an increase from 2.6 mA/cm^2^ at 100 °C to 16.1 mA/cm^2^ at 500 °C). This indicated that the conductivity enhanced with the increase in the annealing temperature. Similarly, the peak current density of these films in the bleaching process shows a similar change (increase from 11.0 mA/cm^2^ at 100 °C to 22.2 mA/cm^2^ at 500 °C). These were attributed to the decrease of *E_g_* and the increase of free electrons. In addition, Figure 4a illustrates that the peak current density of the bleaching process was larger than that of coloring process, which results from the good conductivity of Li_x_WO_3_ [26]. This is related to the decrease of *E_g_* after WO_3_ film coloring.

**Figure 5:**
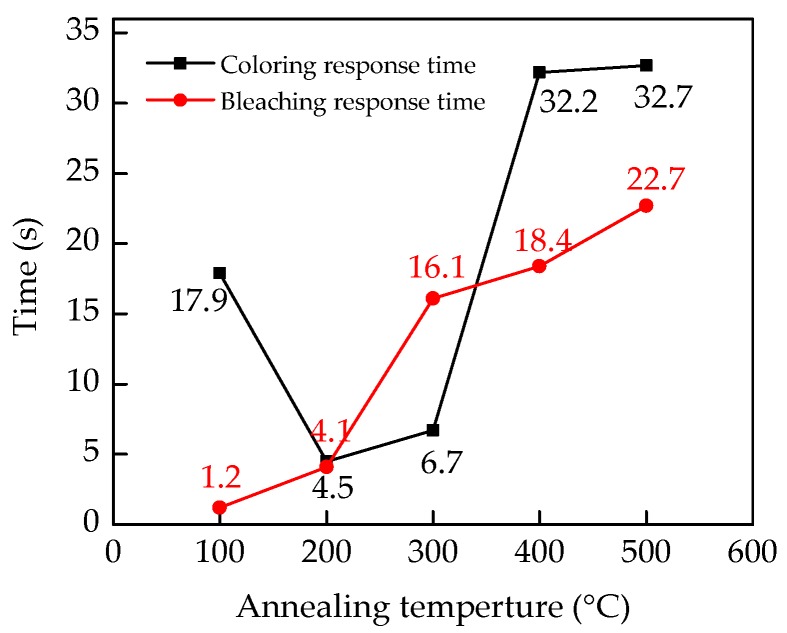
The curves of coloring and bleaching response time versus annealing temperature. The time corresponding to 90% of the total transmittance change is defined as the electrochromic response time.

Figure 4b illustrates an intuitive change of transmittance response curves. The response time is defined by the time corresponding to 90% of the total transmittance change. Figure 5 shows a specific comparison of the response time in the electrochromic test. The curve of the bleaching response time in Figure 5 shows that the bleaching response time increases from 1.2 s to 22.7 s, when the annealing temperature increased. In the bleaching process, the applied voltage drop is mainly across the electrolyte and the Li_x_WO_3_ layer. The extraction of Li^+^ depends largely on the voltage across the Li_x_WO_3_ layer [27]. The *E_g_* of the WO_3_ film at the colored state reduced with the increase in the annealing temperature, which was attributed to the increase in the number of free electrons. In other words, the conductivity enhanced with the increase in annealing temperature. Therefore, the voltage across the Li_x_WO_3_ layer reduced with the increase in annealing temperature, which resulted in the increase of the bleaching response time. However, there was no similar trend in the coloring response time. The influence factors are not only the conductivity of the WO_3_ film, but also the interface barrier of electrolyte-film [28]. The coloring response time increased when the WO_3_ film changed from amorphous into crystalline, which resulted from the decrease in the voltage drop at the WO_3_ layers, due to the increase in the conductivity.The band gap mainly influenced the transmission of the electrons, but the transmission of Li^+^ depended more on the structure of films (such as crystallinity, morphology, etc.) [29]. 

## 4. Conclusions

The effects of the annealing temperature on the *E_g_* of the WO_3_ films were investigated. When the annealing temperature was higher than 400 °C, the crystalline structure of the WO_3_ film changed from amorphous to monoclinic (400 °C), and then to cubic (500 °C). The *E_g_* of the WO_3_ films decreased from 3.62 eV to 3.30 eV when the annealing temperature was increased. In addition, the *E_g_* of the colored WO_3_ films was less than that of the bleached WO_3_ films. The relationship between the *E_g_*, conductivity, and electrochromic response time of the WO_3_ film with different annealing temperatures demonstrates that the conductivity of the WO_3_ film enhanced with the decrease in *E_g_*, while the high conductivity increased the electrochromic response time.
